# Vulnerability of deep neural networks for detecting COVID-19 cases from chest X-ray images to universal adversarial attacks

**DOI:** 10.1371/journal.pone.0243963

**Published:** 2020-12-17

**Authors:** Hokuto Hirano, Kazuki Koga, Kazuhiro Takemoto

**Affiliations:** Department of Bioscience and Bioinformatics, Kyushu Institute of Technology, Iizuka, Fukuoka, Japan; Lingnan University, HONG KONG

## Abstract

Owing the epidemic of the novel coronavirus disease 2019 (COVID-19), chest X-ray computed tomography imaging is being used for effectively screening COVID-19 patients. The development of computer-aided systems based on deep neural networks (DNNs) has become an advanced open source to rapidly and accurately detect COVID-19 cases because the need for expert radiologists, who are limited in number, forms a bottleneck for screening. However, thus far, the vulnerability of DNN-based systems has been poorly evaluated, although realistic and high-risk attacks using universal adversarial perturbation (UAP), a single (input image agnostic) perturbation that can induce DNN failure in most classification tasks, are available. Thus, we focus on representative DNN models for detecting COVID-19 cases from chest X-ray images and evaluate their vulnerability to UAPs. We consider non-targeted UAPs, which cause a task failure, resulting in an input being assigned an incorrect label, and targeted UAPs, which cause the DNN to classify an input into a specific class. The results demonstrate that the models are vulnerable to non-targeted and targeted UAPs, even in the case of small UAPs. In particular, the 2% norm of the UAPs to the average norm of an image in the image dataset achieves >85% and >90% success rates for the non-targeted and targeted attacks, respectively. Owing to the non-targeted UAPs, the DNN models judge most chest X-ray images as COVID-19 cases. The targeted UAPs allow the DNN models to classify most chest X-ray images into a specified target class. The results indicate that careful consideration is required in practical applications of DNNs to COVID-19 diagnosis; in particular, they emphasize the need for strategies to address security concerns. As an example, we show that iterative fine-tuning of DNN models using UAPs improves the robustness of DNN models against UAPs.

## Introduction

Coronavirus disease 2019 (COVID-19) [1] is an infectious disease caused by the coronavirus, called severe acute respiratory syndrome coronavirus 2. The COVID-19 epidemic started from Wuhan, China [2], and has had a severe impact on public health and the economy globally [3]. To reduce the spread of this epidemic, effective screening of COVID-19 patients is required. Thus, positive real-time polymerase chain reaction (PCR) tests are mainly used [4]; however, they are often time consuming and laborious and involve complicated manual processes. Chest radiography, especially chest X-ray computed tomography (CT) imaging, becomes an alternative screening method [5] because patients present abnormalities in chest radiography images, which are a characteristic of those infected with COVID-19 [2, 6]. Moreover, there are advantages to leveraging chest X-ray imaging for COVID-19 screening amid the pandemic in terms of rapid triaging, portability, availability, and accessibility [7]. However, the visual differences in chest X-ray images among COVID-19-associated pneumonia, non-COVID-19 pneumonia, and no pneumonia are subtle; thus, the need for expert radiologists, who are limited in number, forms a bottleneck for diagnoses based on radiography images. To overcome this limitation, computer-aided systems that can aid radiologists in more rapidly and accurately interpreting radiography images to detect COVID-19 cases are highly required [7, 8]; in particular, deep neural networks (DNNs) are often used for this purpose.

DNNs are widely used for image classification, a task in which an input image is assigned a class from a fixed set of classes as well as medical science [9, 10], including diagnoses based on radiography images. Specifically, DNN-based systems can detect subtle visual differences in the images; in particular, a DNN can accurately distinguish bacterial and viral pneumonia in chest X-ray images [11]. Inspired by these previous studies, many researchers have constructed large-scale datasets of chest radiography images on COVID-19 [7, 8, 12, 13] and have proposed DNN-based systems for screening COVID-19 cases from these images [8, 14–17]. However, DNN-based systems in medical science have generally been closed source and unavailable to the research community for deeper understanding and extension. Thus, Wang et al. [7] proposed COVID-Net, a deep convolutional neural network design intended to detect COVID-19 cases from chest X-ray images. COVID-Net is one of the first open-source network designs for COVID-19 detection. As the authors mentioned [7], this study will be leveraged and built upon by both researchers and citizen data scientists to accelerate the development of highly accurate yet practical deep learning solutions for detecting COVID-19 cases and accelerate the treatment of the disease. The COVID-Net models are intended to be used as reference models; in fact, several DNN-based systems [18–20] for detecting COVID-19 cases have already been proposed, inspired by the COVID-Net study.

However, previous studies have poorly evaluated the vulnerabilities in DNNs, although DNNs are known to be vulnerable to adversarial examples [21, 22], which are input images that cause misclassifications by DNNs and are usually generated by adding specific, imperceptible perturbations to original input images that have been correctly classified using DNNs. Adversaries can easily attack open-sourced software, such as COVID-Net because they can access the model parameters and training data; thus, it is important to evaluate the reliability and safety of DNNs against adversarial attacks.

These adversarial attacks may be less useful for adversaries because they are input image dependent (i.e., an individual adversarial perturbation is used such that each input image is misclassified). However, more realistic adversarial attacks have been proposed in recent years. Notably, a single perturbation (called *universal adversarial perturbation*, *UAP*, as they are image agnostic) [23] that can induce DNN failure in most image classification tasks also exists. UAPs are difficult to detect because such perturbations are extremely small and, hence, do not significantly affect data distributions. UAP-based adversarial attacks can be more straightforward to implement by adversaries in real-world environments. A previous study [23] considered only UAPs for non-targeted attacks, which cause misclassification (i.e., a task failure resulting in an input image being assigned an incorrect class). However, we previously extended the algorithm for generating UAPs to enable targeted attacks [24], causing the DNN to classify an input image into a specific class. The existence of adversarial examples questions the generalization ability of DNNs, reduces model interpretability, and limits the applications of deep learning in safety- and security-critical environments [25]. Specifically, vulnerability is a severe problem in medical diagnosis [26]. Thus, it is important to evaluate the vulnerability of the proposed DNN-based systems to adversarial attacks (attacks based on UAPs, in particular) in practical applications. In addition, defense strategies against adversarial attacks (i.e., adversarial defense [22]) are required.

In this study, we focus on the COVID-Net models, which are representative models for detecting COVID-19 cases from chest X-ray images, and aim to evaluate the vulnerability of DNNs to adversarial attacks. Specifically, the vulnerability to non-targeted and targeted attacks, based on UAPs, is investigated. Moreover, adversarial defense is considered; in particular, we evaluate to what extent the robustness of COVID-Net models to non-targeted and targeted UAPs increases using adversarial retraining [23, 27] (i.e., fine-tuning with adversarial images).

## Material and methods

### COVID-Net models

We forked the COVID-Net repository (github.com/lindawangg/COVID-Net) on May 1, 2020, and obtained two DNN models for detecting COVID-19 cases from chest X-ray images: COVIDNet-CXR Small and COVIDNet-CXR Large. Moreover, we downloaded the COVIDx dataset, a collection of chest radiography images from several open-source chest radiography datasets, on May 1, 2020, according to the description in the COVID-Net repository. The chest X-ray images in the dataset were classified into three classes: *normal* (no pneumonia), *pneumonia* (non-COVID-19 pneumonia; e.g., viral and bacterial pneumonia), and *COVID-19* (COVID-19 viral pneumonia). The dataset comprised 13,569 training images (7,966 *normal* images, 5,451 *pneumonia* images, and 152 *COVID-19* images) and 231 test images (100 *normal* images, 100 *pneumonia* images, and 31 *COVID-19* images).

### Universal adversarial perturbations

The UAPs for non-targeted and targeted attacks were generated using simple iterative algorithms [23, 28], whose details are described in [23, 28]. We used the non-targeted UAP algorithm available in the Adversarial Robustness 360 Toolbox (ART) [29] (version 1.0; github.com/IBM/adversarial-robustness-toolbox). The targeted UAP algorithm was implemented by modifying the non-targeted UAP algorithm in the ART in our previous study [24] (github.com/hkthirano/targeted_UAP_CIFAR10).

The algorithms consider a classifier, *C*(***x***), which returns the class or label with the highest confidence score for an input image, ***x***. The algorithm starts with ***ρ*** = **0** (no perturbation) and iteratively updates the UAP, ***ρ***, under the constraint that the *L*_*p*_ norm of the perturbation is equal to or less than a small *ξ* value (i.e., ‖***ρ***‖_*p*_ ≤ *ξ*), by additively obtaining an adversarial perturbation for an input image, ***x***, which is randomly selected from an input image set, ***X***, without replacement. These iterative updates continue until the number of iterations reaches a maximum *i*_max_.

We used the fast gradient sign method (FGSM) [21] to obtain an adversarial perturbation for the input image, instead of the original UAP algorithm [23], which uses the DeepFool method [30]. This is because FGSM is used for both non-targeted and targeted attacks, and DeepFool requires a higher computational cost than FGSM and only generates a non-targeted adversarial example for the input image. FGSM generates the adversarial perturbation, $\hat{\rho }$, for ***x*** using gradient ∇_*x*_*L*(***x***, *y*) of the loss function at the specified image ***x*** and class *y* with respect to the pixels [21]. For the *L*_∞_ norm, a non-targeted perturbation that causes misclassification is computed as $\hat{\rho }=\epsilon \cdot \text{sign}(\nabla_{x}L(x,C(x))$), whereas a targeted perturbation that causes *C* classification of an image ***x*** into class *y* is obtained as $\hat{\rho }=-\epsilon \cdot \text{sign}(\nabla_{x}L(x,y))$, where ϵ (> 0) is the attack strength. For the *L*_1_ and *L*_2_ norms, a non-targeted perturbation is computed as $\hat{\rho }=\epsilon \cdot \nabla_{x}L(x,C(x))/∥\nabla_{x}L(x,C(x)){∥}_{p}$, whereas a targeted perturbation is obtained as $\hat{\rho }=-\epsilon \cdot \nabla_{x}L(x,y)/{∥\nabla_{x}L(x,y)∥}_{p}$.

In the algorithms, FGSM is performed based on the output *C*(***x*** + ***ρ***) of the classifier for the perturbed image ***x*** + ***ρ***, at each iteration step. For non-targeted (targeted) attacks, an adversarial perturbation, $\hat{\rho }$, for ***x*** + ***ρ*** is obtained using the FGSM if *C*(***x*** + ***ρ***) = *C*(***x***) · (*C*(***x*** + ***ρ***) ≠ *y*). After generating the adversarial example (i.e., $x_{\text{adv}}\leftarrow x+\rho +\hat{\rho }$) at this step, the perturbation ***ρ*** is updated if *C*(***x***_adv_) ≠ *C*(***x***) (*C*(***x***_adv_) = *y*) for non-targeted (targeted) attacks. When updating ***ρ***, a projection function project, (***x***, *p*, *ξ*), is used to satisfy the constraint that ‖***ρ***‖_*p*_ ≤ *ξ*: ***ρ*** ← project(***x***_adv_ − ***x***, *p*, *ξ*), where project(***x***, *p*, *ξ*) = arg min_*x*′_‖***x*** − ***x***′‖_2_ subject to ‖***ρ***‖_*p*_ ≤ *ξ*.

The non-targeted and targeted UAPs were generated using 13,569 training images in the COVIDx dataset. Parameter ϵ was set to 0.001; the cases where *p* = 2 and ∞ were considered. Meanwhile, parameter *ξ* was determined based on the ratio *ζ* of the *L*_*p*_ norm of the UAP to the average *L*_*p*_ norm of an image in the COVIDx dataset. Cases in which *ζ* = 1% and 2% (i.e., almost imperceptible perpetuations) were considered. The average *L*_∞_ and *L*_2_ norms were 237 and 32,589, respectively; *i*_max_ was set to 15.

To compare the performance of the generated UAPs with that of random controls, we also generated random vectors (random UAPs) sampled uniformly from the sphere of a specified radius [23].

### Vulnerability evaluation

To evaluate the vulnerability of the DNN models to UAPs, we used the fooling rate, *R*_*f*_, and targeted the attack success rate, *R*_*s*_, of non-targeted and targeted attacks, respectively. The *R*_*f*_ of an image set is defined as the proportion of images that were not classified into their associated actual labels to all images in the set. The *R*_*s*_ of an image set is the proportion of adversarial images classified into the target class to all images in the set. Additionally, we obtained the confusion matrices to evaluate the change in prediction owing to the UAPs for each class (infection type).

### Adversarial retraining

We performed adversarial retraining to increase the robustness of the COVID-Net models to UAPs [23, 27]; in particular, the models were fine-tuned with adversarial images, and the procedure was described in a previous study [23]. A brief description is provided below. 1) Ten UAPs against a DNN model were generated using the algorithm (for generating a non-targeted or targeted UAP) (see Materials and methods section) with the (clean) training image set. 2) A modified training image set was obtained by randomly selecting half of the training images and combining them with the rest, where each image was perturbed by a UAP randomly selected from 10 UAPs. 3) The model was fine-tuned by performing five extra epochs of training on the modified training image set. 4) A new UAP (against the fine-tuned model) was generated using the algorithm with the training image set. 5) *R*_*f*_ and *R*_*s*_ of the UAP for the test images were then computed. Steps 1)–5) were repeated five times.

## Results

### Performance of COVID-Net models

The test accuracies of the COVIDNet-CXR Small and COVIDNet-CXR Large models were 92.6% and 94.4%, respectively, and their training accuracies were 95.8% and 94.1%, respectively. As shown in the COVID-Net study [7], we also confirmed that the COVID-Net models achieved good accuracies.

### Vulnerability to non-targeted universal adversarial perturbations

However, we found that both COVIDNet-CXR Small and COVIDNet-CXR Large models were vulnerable to non-targeted UAPs (Table 1). Specifically, the fooling rate, *R*_*f*_, of the UAPs with *ζ* = 1% for the test image set was 81.0% at most. A higher *ζ* led to a higher *R*_*f*_. We observed that the *R*_*f*_ of the UAP with *ζ* = 2% for the test image set was between 85.7% and 87.4%. Furthermore, the random UAPs with *ζ* = 2% misclassified the models; specifically, their *R*_*f*_ were up to 22.1%. The change in *R*_*f*_ did not exhibit significant dependence on the norm types (*p* = 2 or ∞). The difference in *R*_*f*_ for the test image set between *p* = 2 and *p* = ∞ was up to 7%, the model and the other parameters being equal. *R*_*f*_ of the UAP against the COVIDNet-CXR Small model was lower than that of the COVIDNet-CXR Large model in the case of *ζ* = 1%, the model and the other parameters being equal; however, no remarkable difference in *R*_*f*_ between these models was observed in the case of *ζ* = 2%. The *R*_*f*_ of the training image set was higher than that of the test image set because the UAPs were generated based on the training image set.

**Table 1:** Fooling rates *R*_*f*_ (%) of non-targeted UAPs against the COVID-Net models.

*p*	*ζ*	COVIDNet-CXR Small		COVIDNet-CXR Large	
		Training	Test	Training	Test
2	1%	61.4 (1.3)	58.0 (0.4)	90.0 (2.5)	81.0 (3.9)
	2%	98.5 (12.6)	87.4 (16.0)	97.4 (17.9)	85.7 (22.1)
∞	1%	70.8 (1.0)	64.9 (1.3)	84.8 (2.0)	77.1 (3.5)
	2%	98.5 (9.4)	87.4 (13.4)	97.4 (14.3)	85.7 (19.9)

The *R*_*f*_ of the training and test images are presented. The values in the brackets indicate *R*_*f*_ random UAPs (random controls).

**Fig 1:**
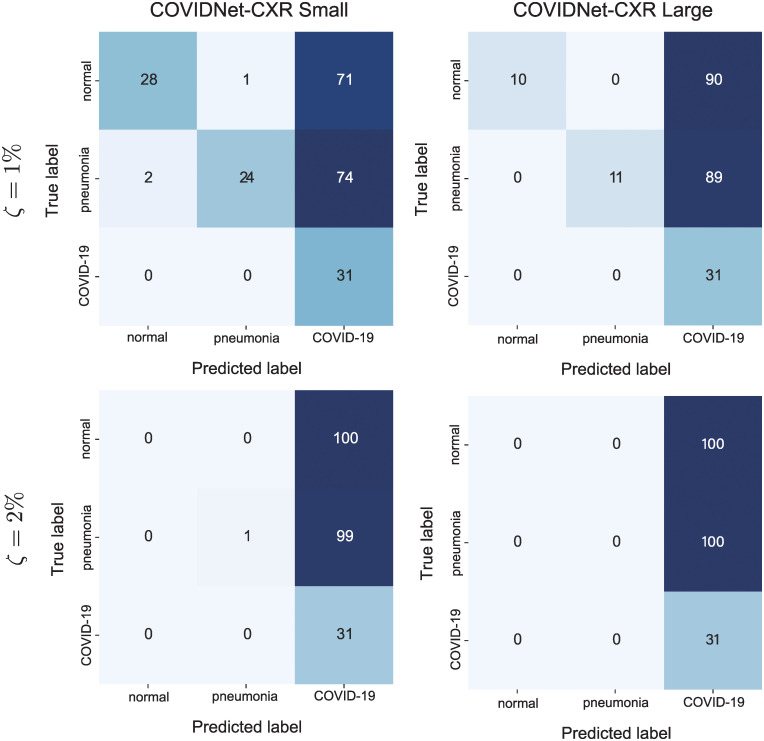
Confusion matrices for the COVID-Net models attacked using the non-targeted UAPs on the test images. *p* = ∞. Left and right panels represent the COVIDNet-CXR Small and COVIDNet-CXR Large models, respectively. The top and bottom panels indicate *ζ* = 1% and *ζ* = 2%, respectively.

Owing to non-targeted UAPs, the models classified most images into *COVID-19*. Fig 1 shows the confusion matrices for the COVID-Net models attacked using non-targeted UAPs with *p* = ∞. For the UAPs with *ζ* = 1%, the COVIDNet-CXR Small model classified >70% of the *normal* and *pneumonia* test images into *COVID-19*. Moreover, the COVIDNet-CXR Large model classified approximately 90% of the *normal* and *pneumonia* images into *COVID-19*. For a higher *ζ*, this tendency was more significant. In particular, the COVIDNet-CXR Small and Large models evaluated almost all *normal* and *pneumonia* test images as COVID-19 cases when *ζ* = 2%. Additionally, the tendency of adversarial images to be classified into *COVID-19* was observed when considering UAPs with *p* = 2 and the training image set.

**Fig 2:**
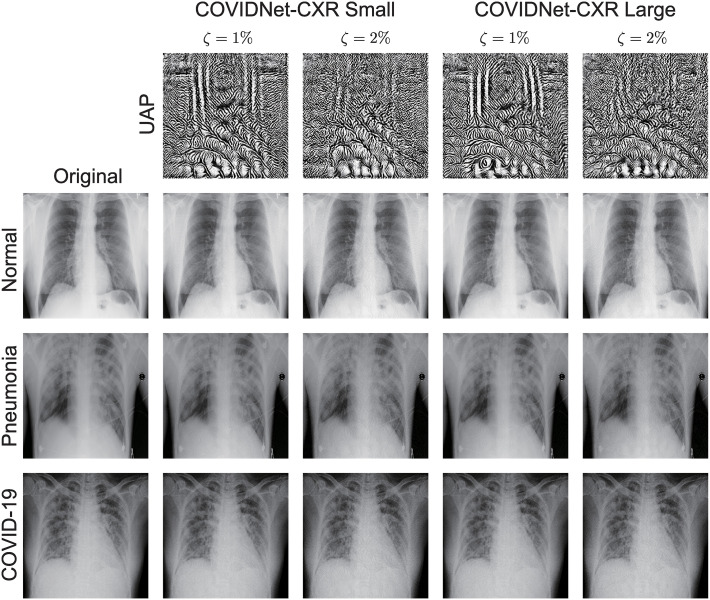
Non-targeted UAPs with *p* = ∞ against the COVID-Net models and their adversarial images. UAPs (top panels) with *ζ* = 1% and *ζ* = 2% are shown. The models correctly classified the original images (left panels) into their actual labels. The predicted labels of all adversarial images are of *COVID-19*. Note that the UAPs are emphatically displayed for clarity; in particular, each UAP is scaled by a maximum of 1 and a minimum of 0.

The non-targeted UAPs with *ζ* = 1% and *ζ* = 2% were almost imperceptible. Fig 2 shows the non-targeted UAPs *p* = ∞ against the COVID-Net models and their adversarial images. The models classified the original X-ray images (left panels in Fig 2) and correctly predicted their actual classes; however, they evaluated all adversarial images as COVID-19 cases owing to the non-targeted UAPs. Similarly, the non-targeted UAPs *p* = 2 were almost imperceptible.

### Vulnerability to targeted universal adversarial perturbations

Furthermore, we found that both the COVIDNet-CXR Small model (Table 2) and COVIDNet-CXR Large model (Table 3) were vulnerable to targeted UAPs. Subsequently, we considered the effect of the targeted attacks using UAPs in each class: *normal*, *pneumonia*, and *COVID-19*. When *ζ* = 1%, the targeted attack success rates *R*_*s*_ for the test images were between approximately 60% and 85% and between approximately 55% and 95% for the COVIDNet-CXR Small and Large models, respectively. Conversely, the *R*_*s*_ of the training images was between approximately 65% and 90% and between approximately 55% and 90%. Meanwhile, the *R*_*s*_ of the UAP with *p* = 2 was higher than that of the UAP with *p* = ∞, the model, and the other parameters being equal. Moreover, no remarkable difference in the *R*_*s*_ was observed between the target classes; however, the *R*_*s*_ of the target attacks to *COVID-19* were relatively high in the COVIDNet-CXR Large model. Thus, a higher *ζ* led to a higher *R*_*s*_. When *ζ* = 2%, the *R*_*s*_ values for both the training and test images were approximately 100%, regardless of the target classes. For the targeted attacks to *normal* and *pneumonia*, the *R*_*s*_ of random UAPs for the test images were also relatively high; in particular, they were between approximately 35% and 45% and between approximately 30% and 45% for the COVIDNet-CXR Small model and COVIDNet-CXR Large model, respectively.

**Table 2:** Targeted attack success rate *R*_*s*_ (%) of targeted UAPs against the COVIDNet-CXR Small model to each target class.

*p*	*ζ*	*Normal*		*Pneumonia*		*COVID-19*	
		Training	Test	Training	Test	Training	Test
2	1%	88.1 (60.5)	78.4 (46.3)	76.7 (37.5)	71.4 (41.6)	68.1 (1.9)	74.0 (12.1)
	2%	99.4 (54.4)	97.8 (39.0)	99.4 (33.0)	98.7 (35.9)	100 (12.6)	99.1 (25.1)
∞	1%	79.5 (60.7)	64.9 (45.9)	66.5 (37.5)	61.9 (41.6)	78.8 (1.8)	84.0 (12.6)
	2%	98.7 (56.3)	96.1 (39.4)	99.5 (34.1)	98.3 (37.7)	100 (9.5)	100 (22.9)

The *R*_*s*_ for the training and test images are shown in Table 2. The values in brackets are *R*_*s*_ random UAPs (random controls).

**Table 3:** Targeted attack success rates *R*_*s*_ (%) of targeted UAPs against the COVIDNet-CXR Large model to each target class.

*p*	*ζ*	*Normal*		*Pneumonia*		*COVID-19*	
		Training	Test	Training	Test	Training	Test
2	1%	85.2 (58.9)	71.4 (44.2)	72.6 (37.0)	66.2 (39.0)	92.4 (4.0)	95.2 (16.9)
	2%	99.2 (50.7)	98.3 (34.6)	99.5 (30.6)	98.7 (32.9)	100 (18.7)	100 (32.5)
∞	1%	71.0 (59.2)	56.7 (44.2)	55.4 (37.0)	53.2 (40.3)	88.4 (3.7)	92.2 (15.6)
	2%	97.9 (52.7)	93.9 (35.9)	99.4 (32.3)	98.3 (33.8)	100 (14.9)	100 (30.3)

The *R*_*s*_ for the training and test images are shown in Table 3. The values in brackets are *R*_*s*_ random UAPs (random controls).

**Fig 3:**
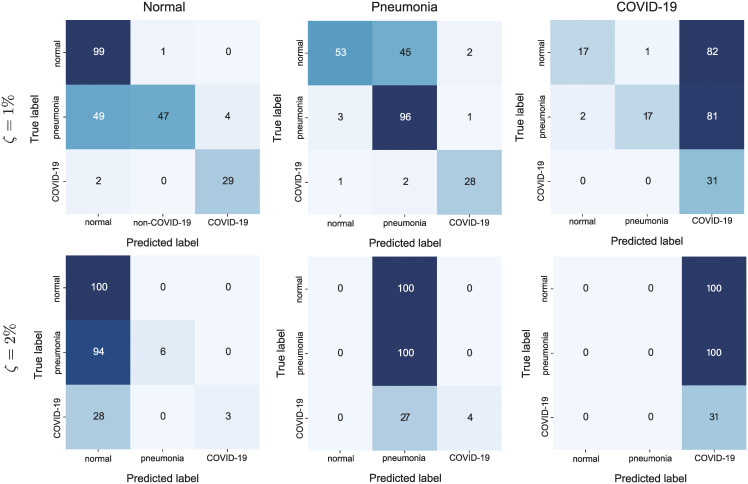
Confusion matrices for the COVIDNet-CXR Small model attacked with the targeted UAPs with *p* = ∞ on the test images. The left, middle, and right panels represent the targeted classes: *normal*, *pneumonia*, and *COVID-19*, respectively. The top and bottom panels indicate *ζ* = 1% and *ζ* = 2%, respectively.

It was difficult to classify the *COVID-19* images into another targeted class (*normal* or *pneumonia*) when the UAPs were relatively weak (i.e., *ζ* = 1%). Fig 3 shows the confusion matrices for the COVIDNet-CXR Small model attacked using targeted UAPs with *p* = ∞. For both targeted attacks to *normal* and *pneumonia*, the model correctly predicted almost all *COVID-19* images as COVID-19 cases, despite the targeted attacks. Conversely, approximately 50% of *normal* (*pneumonia*) images were classified as targeted class *pneumonia* (*normal*). However, for a higher *ζ* (i.e., *ζ* = 2%), the targeted attacks of the *COVID-19* images were successful; in particular, almost all *COVID-19* images were classified into the target class (*normal* or *pneumonia*) because of the UAP. The classification of the images into COVID-19 using targeted UAPs was easier than that into the other classes. Owing to the UAP with *ζ* = 1%, the model judged approximately 80% of *normal* and *pneumonia* images as COVID-19 cases, respectively. Similar tendencies were observed in the COVIDNet-CXR Large model for targeted UAPs with *p* = 2 and on the training image set.

**Fig 4:**
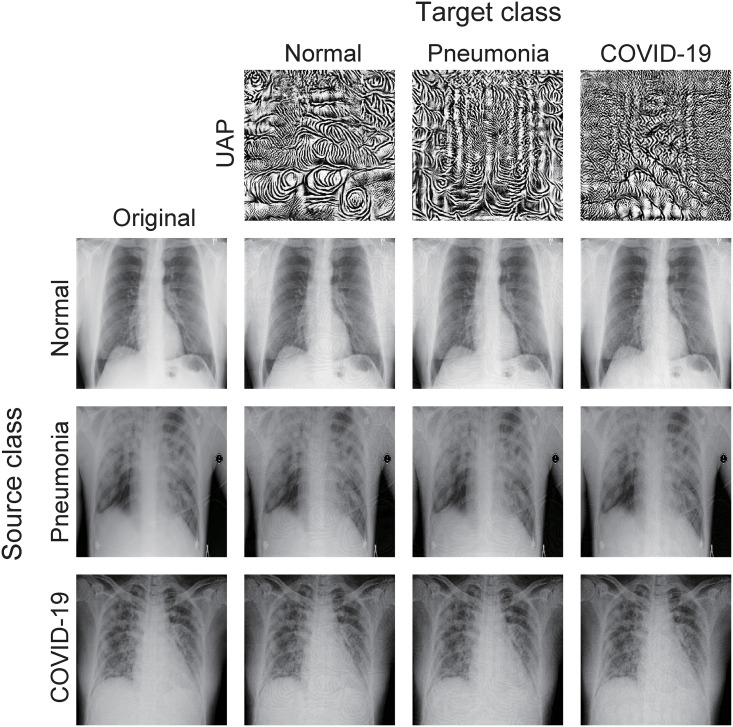
Targeted UAPs (top panel) with *ζ* = 2% and *p* = ∞ against the COVIDNet-CXR Small model and their adversarial images. Note that UAPs are emphatically displayed for clarity; in particular, each UAP is scaled by a maximum of 1 and a minimum of 0.

The targeted UAPs were also almost imperceptible. Fig 4 shows the targeted UAPs with *p* = ∞ and *ζ* = 2% against the COVIDNet-CXR Small model and their adversarial images. The model classified the original images (left panels in Fig 4) and correctly predicted their actual classes (source classes); however, it classified the adversarial images into each target class because of the targeted UAPs. The UAPs with *ζ* = 1% were also imperceptible. Additionally, imperceptibility was confirmed in the UAPs with *p* = 2 and those against the COVIDNet-CXR Large model.

### Effect of adversarial retraining

**Fig 5:**
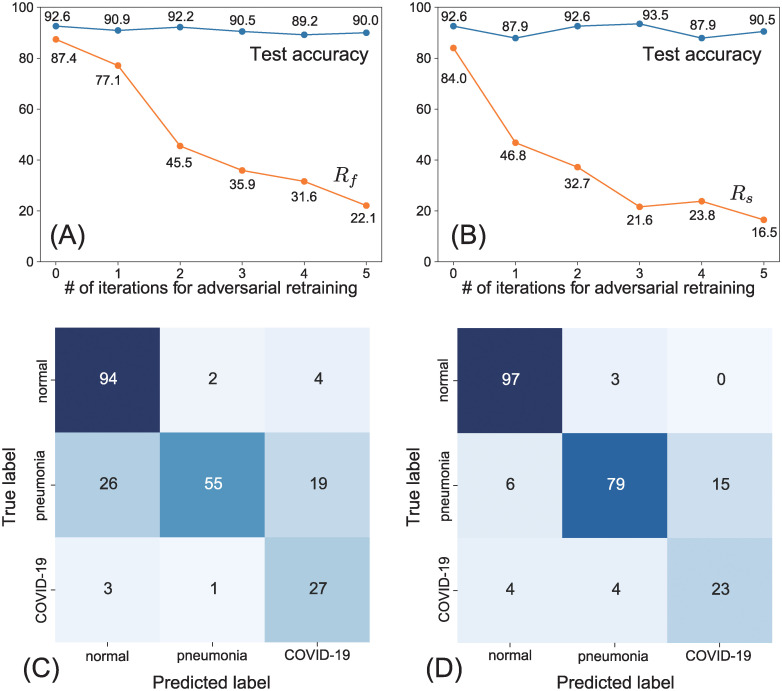
Effect of adversarial retraining on the robustness to UAPs with *p* = ∞ against the COVIDNet-CXR Small model. Scatter plots of (A) the fooling rate, *R*_*f*_ (%), for non-targeted UAPs with *ζ* = 2% versus the number, *N*_*i*_, of iterations for adversarial retraining and (B) the targeted attack success rate, *R*_*s*_ (%), of targeted UAPs with *ζ* = 1% to *COVID-19* versus *N*_*i*_. Here, *R*_*f*_ and *R*_*s*_ are for the test images. The accuracies (%) on the set of clean test images are also shown. The confusion matrices for the fine-tuned models were obtained after five iterations of adversarial retraining using the (C) non-targeted UAPs and (D) targeted UAPs. Note that these confusion matrices belong to the fine-tuned models attacked using non-targeted and targeted UAPs, respectively.

Adversarial retraining is often used to avoid adversarial attacks. In this study, we investigated the extent to which adversarial retraining increases the robustness of the COVIDNet-CXR Small model to non-targeted and targeted UAPs with *p* = ∞. Adversarial retraining did not affect the test accuracy in either non-targeted or targeted cases; specifically, the accuracy on the (clean) test images remained constant at approximately 90% (Fig 5A and 5B).

For non-targeted attacks using UAPs with *ζ* = 2%, *R*_*f*_ for the test images declined with the iterations for adversarial retraining; in particular, it was 22.1% after five iterations (Fig 5A). The confusion matrix (Fig 5C) for the fine-tuned model obtained after five iterations indicates that the *normal* and *COVID-19* images were almost correctly classified despite the non-targeted UAPs. However, 45% of the *pneumonia* images were still misclassified.

For targeted attacks to *COVID-19* using UAPs with *ζ* = 1%, the *R*_*s*_ for the test images decreased with the iterations for adversarial retraining (Fig 5B); specifically, it was 16.5% after five iterations. The confusion matrix (Fig 5D) for the fine-tuned model obtained after five iterations indicates that the *normal* and *COVID-19* images were almost correctly classified despite the targeted UAPs. However, 15% of the *pneumonia* images were still misclassified as *COVID-19*.

## Discussion

The COVID-Net models were vulnerable to small UAPs; moreover, they were slightly less robust to random UAPs. The results indicated that the DNN-based systems were easy to mislead. Adversaries can result in failing the DNN-based systems at lower costs (i.e., using a single perturbation); specifically, they do not need to consider the distribution and diversity of input images when attacking the DNNs using UAPs, as UPAs are image agnostic. Considering that vulnerability to UAPs is observed in various DNN architectures [23, 24], they are expected to exist universally in DNN-based systems for detecting COVID-19 cases.

For non-targeted attacks with UAPs, the COVID-Net models predicted most of the chest X-ray images as COVID-19 cases because of the UAPs (Fig 1), although the UAPs were almost imperceptible (Fig 2). This result is consistent with the tendency of DNN models to classify most inputs into a few specific classes because of non-targeted UAPs (i.e., existence of dominant labels in non-targeted attacks based on UAPs) [23]. Moreover, this indicates that the models provide false positives in COVID-19 diagnosis, which may cause unwanted mental stress to patients and complicate the estimation of the number of COVID-19 cases. The dominant label of COVID-19 observed in this study may be because the COVIDx dataset was imbalanced. The images in *COVID-19* were predominantly fewer than those in *normal* and *pneumonia* cases. The algorithm considers maximizing the fooling rate; thus, a relatively large fooling rate is achieved when all inputs are classified into *COVID-19* because of UAPs. In addition, the observed dominant label may be because the losses were computed by weighting the *COVID-19* class to consider the imbalanced dataset. The decision for the *COVID-19* class might be more susceptible to changes in pixel values than that for the other classes.

The relatively easy targeted attacks on *COVID-19* (Fig 3) may be because *COVID-19* was the dominant label. Moreover, targeted attacks to *normal* and *pneumonia* were possible, despite almost imperceptible UAPs (Fig 4). The results imply that adversaries can control DNN-based systems, which may lead to security concerns. The targeted attacks cause both false positives and negatives, and thus, can be used to adjust the number of COVID-19 cases. Moreover, they may affect individual and social awareness of COVID-19 (e.g., voluntary restraint and social distancing). These may lead to problems in terms of public health (i.e., minimizing the spread of the pandemic) and the economy. More generally, complex classifiers, including DNNs, are currently used for high-stake decision making in healthcare; however, they can potentially cause catastrophic harm to the society because they are often difficult to interpret [31].

The COVID-Net models, with tailored network architecture, seem to be more vulnerable to adversarial attacks than representative DNN models (e.g., VGG [32] and ResNet [33] models) for classifying ideal natural images (e.g., CIFAR-10 [34] and ImageNet datasets [35]). For these representative DNNs, UAPs with *ζ* = 5% and higher are required to achieve >80% success rates for non-targeted and targeted attacks [23, 28]. Conversely, for the COVID-Net models, UAPs with *ζ* = 2% achieved >85% and >90% success rates for the non-targeted and targeted attacks, respectively. This result implies several possible reasons that caused the vulnerability of COVID-Net models. For example, the variance (visual difference) in chest X-ray images is much less than that in natural images. In this case, data points may aggregate around decision boundaries, indicating that the outputs of the DNN models are susceptible to changes in pixel values. As a result, adversarial examples are easy to generate. In addition, the fact that adversarial vulnerability of DNNs is known to increase with input dimension [36] may be one of the causes.

The UAPs used in this study are a type of white-box attack, which assumes that adversaries can access the model parameters (the gradient of the loss function, in this case) and training images; thus, they are security threats for open-source software projects, such as COVID-Net. A simple solution to prevent these adversarial attacks is to make DNN-based systems closed-source and publicly unavailable; however, this conflicts with the purpose of accelerating the development of computer-based systems for detecting COVID-19 cases and COVID-19 treatment. An alternative may be to consider black-box systems, such as closed application programming interfaces (APIs) and closed-source software in which only queries on inputs are allowed and outputs are accessible. Such closed APIs are better because they are at least publicly available. However, it is possible that APIs are vulnerable to adversarial attacks. This is because UAPs have generalizability [23] (i.e., UAPs for a DNN can mislead another DNN). That is, adversarial attacks on black-box DNN-based systems may be possible using the UAPs generated based on white-box DNNs. Moreover, several methods for adversarial attacks on black-box DNN-based systems, which estimate adversarial perturbations using only model outputs (e.g., confidence scores), have been proposed [37–39].

Therefore, defense strategies against adversarial attacks should be considered. A simple defense strategy is to fine-tune DNN models using adversarial images [22, 23, 27]. In fact, we demonstrated that iterative fine-tuning of a DNN model using UAPs improved the robustness of the DNN model to non-targeted and targeted UAPs (Fig 5). However, the iterative fine-tuning method required high computational costs, and it did not perfectly avoid vulnerability to UAPs. In addition, several methods breaching defenses using adversarial retraining have already been proposed [27]. Alternatively, dimensionality reduction (e.g., principle component analysis), distributional detection (e.g., maximum mean discrepancy), and normalization detection (e.g., dropout randomization) may be useful for adversarial defenses; however, adversarial examples are not easily detected using these approaches [27]. Defending against adversarial attacks is a cat-and-mouse game [26]; thus, it may be difficult to completely avoid security concerns caused by adversarial attacks. However, the development of methods for defending against adversarial attacks has advanced. For example, detecting adversarial attack-based robustness to random noise [40], the use of a discontinuous activation function that purposely invalidates the DNN’s gradient at densely distributed input data points [41], and DNNs for purifying adversarial examples [42] may help reduce the concerns.

In conclusion, we demonstrated the vulnerability of DNNs for detecting COVID-19 cases to non-targeted and targeted attacks based on UAPs. However, many studies have developed DNN-based systems for detecting COVID-19 while ignoring the vulnerability. Our findings emphasize that careful consideration is required in developing DNN-based systems for detecting COVID-19 cases and their practical applications. Facile applications of DNNs to COVID-19 detection could lead to problems in terms of public health and the economy. Our study is the first to show the vulnerability of DNNs for COVID-19 detection and to alert such facile applications of DNNs. The code used in this study is available from our GitHub repository: github.com/hkthirano/UAP-COVID-Net. The chest X-ray images used in this study are publicly available online (see github.com/lindawangg/COVID-Net/blob/master/docs/COVIDx.md for details).
